# Praising others differently: neuroanatomical correlates to individual differences in trait gratitude and elevation

**DOI:** 10.1093/scan/nsy093

**Published:** 2018-10-23

**Authors:** Guanmin Liu, Guang Zeng, Fei Wang, Pia Rotshtein, Kaiping Peng, Jie Sui

**Affiliations:** 1 Department of Psychology, Tsinghua University, Beijing, China; 2 School of Psychology, University of Birmingham, Birmingham, UK; 3 Department of Psychology, University of Bath, Bath, UK

**Keywords:** gratitude, elevation, fusiform gyrus, posterior superior temporal sulcus, dorsolateral prefrontal cortex

## Abstract

Differing from basic emotions such as happiness, gratitude and elevation are important other-praising emotions. Previous behavioral studies have established that these complex emotions differ from each other; however, it remains under-investigated whether proneness to these emotions have common or distinct neuroanatomical correlates. Here we used voxel-based morphometry to identify the common and distinct neuroanatomical correlates of trait (i.e. proneness to) gratitude and elevation. We used the Gratitude Questionnaire-6 and the trait elevation scale to measure these affective traits. We demonstrated that trait gratitude was positively correlated with gray matter volume (GMV) in the left cerebellum extending to fusiform gyrus, and also the right middle occipital gyrus (MOG) extending to posterior superior temporal sulcus (pSTS) and temporoparietal junction (TPJ), while trait elevation was negatively correlated with GMV in the left dorsolateral prefrontal cortex. While controlling each other, all the regions still held significant, except the right MOG and pSTS/TPJ. The results indicate that there are distinct neuroanatomical correlates for proneness to gratitude and elevation, while the evidence is mixed that pSTS/TPJ may be the common correlates for them. The implications of these findings are discussed.

## Introduction

Previous research of emotions has mainly focused on basic emotions (e.g. happiness, fear) proposed by Ekman and Friesen (1971). Beyond these emotions, however, it has been found that complex social emotions are essential to human daily activities (Schachter and Ekman, 2005). Among them, other-praising or self-transcendent emotions, such as gratitude, admiration and elevation, are foundational to the complex social processes that define the human species (Algoe and Haidt, 2009; Haidt and Morris, 2009; Stellar *et al.*, 2017).

According to Fredrickson (1998), all positive emotions, including other-praising emotions like gratitude (Fredrickson, 2004) and elevation (Haidt, 2000), serve to broaden an individual’s momentary thought–action repertoire, which in turn has the effect of building that individual’s physical, intellectual and social resources. An abundance of behavioral studies has demonstrated that other-praising emotions as well as other positive emotions facilitate prosocial behaviors (Isen, 1987; Bartlett and DeSteno, 2006; Schnall *et al*., 2010). Researchers have shown that though sharing some common characteristics, other-praising emotions are distinct from other positive emotions, especially the more basic ones (Algoe and Haidt, 2009; Stellar *et al*., 2017). It has been argued that other-praising and self-transcendent emotions should be more strongly bounded by group membership, improve social relationships and be more frequently strategically displayed than other positive emotions (Stellar *et al.*, 2017). Among these emotions, gratitude and elevation are most similar to each other, as both of them are other-focused positive-valenced moral emotions (Tangney *et al.*, 2007). Both gratitude and elevation showed stronger effect on facilitating prosocial behavior than the more basic positive emotion (Bartlett and DeSteno, 2006; Silvers and Haidt, 2008).

It has been found that gratitude and elevation are distinct from commonly studied positive emotions, such as joy (Algoe and Haidt, 2009; Siegel *et al*., 2014; Stellar *et al*., 2017). Gratitude is the emotion experienced when one affirms that something good has happened to them, and they recognize that someone else is largely responsible for this benefit (Watkins, 2014). An abundance of research has demonstrated that gratitude facilitates prosocial behavior (Bartlett and DeSteno, 2006; DeSteno *et al*., 2010; Grant and Gino, 2010). More importantly, this prosocial effect is stronger compared to that of amusement, a more basic positive emotion (Bartlett and DeSteno, 2006). In addition, gratitude motivates improved relationships with benefactors (Algoe and Haidt, 2009), and serves the function of strengthening a relationship with a responsive interaction partner (Algoe, 2012; Algoe *et al*., 2008). Previous studies have shown that the benevolent intention is the essence of gratitude induction (McConnell, 1993; Yu *et al*., 2017).

In contrast, elevation is conceptualized as the warm and uplifting feeling that people experience when they see unexpected acts of human goodness, kindness and compassion (Haidt, 2000). Because elevation is often induced by witnessing positive moral or virtuous behavior, it is also called moral elevation (Silvers and Haidt, 2008; Englander *et al*., 2012; Palmer *et al*., 2013; Amoyal, 2014; Pohling and Diessner, 2016) and sometimes called admiration of virtue (Immordino-Yang *et al*., 2009). Physically, elevation elicits physical responses, such as warm feeling in one’s chest, pleasant sensations in the body and a lump in throat (Algoe and Haidt, 2009). Behaviorally, elevation encourages the observer to mimic the behavior of the virtuous role model, and thus leads to prosocial behaviors (Freeman *et al*., 2009; Landis *et al*., 2009; Schnall *et al*., 2010). Moreover, this prosocial effect is also stronger compared to that of amusement. For example, Silvers and Haidt (2008) found that nursing mothers having watched a video inducing elevation showed more nurturing behavior towards their infants than those having watched an amusing video.

Furthermore, Algoe and Haidt (2009) showed that gratitude and elevation are distinct from each other in terms of the motivational and behavioral consequences induced. Gratitude motivates the beneficiary to repay benefactor, praise benefactor publicly, and to cultivate closer relationship with benefactor. In contrast, elevation motivates the witness to do good deeds, emulate and become a good person, and to be open to others in general. Though both gratitude and elevation facilitate prosocial behaviors, gratitude is more relationship-focused and promotes repayment to the benefactor, while elevation is more morality-focused and promotes altruism towards people other than the virtuous role model. This was supported by a recent study (Siegel *et al*., 2014) showing that the morality of the recipient of a potential donation predicted more strongly the donation choice of people who are elevated than those feeling grateful, indicating that people are more sensitive to the morality judgement of the beneficiary when they feel elevation than gratitude. Similarly, elevation was also shown to decrease permissiveness for deontological violations in moral judgment (Strohminger *et al*., 2011).

However, most of the studies compared gratitude and elevation on the state level. As suggested by Rosenberg (1998), affects can be defined at two levels–state and trait. State affect is acute and brief psychophysiological changes in response to a meaningful situation, while trait affect is a stable predisposition towards certain types of emotional responses. This study was to investigate the common and distinct neuroanatomical correlates of gratitude and elevation on the trait level.

Some research has been carried out to investigate the neural basis of gratitude or elevation (Zahn *et al.*, 2008; Immordino-Yang *et al.*, 2009; Englander *et al.*, 2012; Fox *et al.*, 2015; Kini *et al.*, 2016; Yu *et al.*, 2017; Yu *et al.*, 2018). For example, Zahn *et al.*(2008) compared the neural basis of gratitude, pride, guilt and indignation. They found that all four social values activated superior anterior temporal lobe (aTL), while gratitude additionally activated hypothalamus. Although shedding important light into our understanding about the neural mechanism underlying these emotions, most of the previous studies focused on the state level of gratitude and elevation. The neuroanatomical correlates of individual differences in trait elevation remain unclear. Up to now, only two studies (Zahn *et al.*, 2013; Yang *et al.*, 2018) have investigated the neurostructural correlates of trait gratitude. Zahn *et al.*(2013) found that proneness to gratitude was associated with greater right inferior temporal volume, disruption of which is associated with both acquired (Barton, 2008) and developmental prosopagnosia (Dinkelacker *et al.*, 2011), indicating visuo-spatial memories and mental models are required to support higher proneness to gratitude. However, the study used a measure developed for an fMRI study (Zahn *et al.*, 2008) rather than one specifically developed to measure individual differences. Its reproducibility needs to be examined. A recent study (Yang *et al.*, 2018) applied a more widely-used measure, the Gratitude Questionnaire-6 (GQ-6; McCullough *et al.*, 2002), and found that gratitude was negatively correlated with the lateral rostral prefrontal cortex volume. However, because the objective of that study was to investigate the neurostructural correlates of maternal emotional warmth rather than gratitude, it did not provide whole-brain analysis results for the neural correlates of gratitude, failing to provide evidence for the reproducibility of Zahn *et al.*’s (2013) results. In addition, both studies did not include trait elevation, whether there were distinct brain structures supporting trait gratitude or trait elevation remained unknown. This was tested here using voxel-based morphormetry (VBM) with a large sample of adults.

In summary, the present study aimed to explore whether there are common and distinct neurostructural correlates of individual differences in trait gratitude and elevation. Specifically, we aimed to investigate whether previous findings in the relationship between gratitude-proneness and gray matter volume (GMV) in posterior cortex (Zahn *et al.*, 2013) could be replicated, especially inferior temporal gyrus and another region closely associated with face processing, i.e. fusiform gyrus. In addition, we aimed to examine whether this relationship is distinctive to trait gratitude (compared to elevation). Because no VBM study has ever been conducted on elevation and the only two fMRI studies on elevation provided inconsistent results (Immordino-Yang *et al.*, 2009; Englander *et al.*, 2012), it is an exploratory study. Our main expectations are that trait elevation would be associated with regions critical to moral judgment because it is involved in virtue recognition (admiration for virtue), including ventromedial prefrontal cortex (VMPFC) and dorsolateral prefrontal cortex (DLPFC; Moll *et al.*, 2005; Greene, 2007, 2009; Moll and de Oliveira-Souza, 2007a, 2007b; Moll *et al.*, 2008; Zahn *et al.*, 2008). On the other hand, based on previous fMRI studies (Zahn *et al.*, 2008; Fox *et al.*, 2015; Kini *et al.*, 2016; Wang *et al.*, 2017; Yu *et al.*, 2017; Yu *et al.*, 2018), we hypothesized that trait gratitude and trait elevation would be related to regions associated with reward processing, including medial prefrontal cortex (MPFC) and hypothalamus and also regions associated with intentionality inference, including MPFC, posterior superior temporal sulcus (pSTS), temporoparietal junction (TPJ) and temporal pole/aTL (Zahn *et al.*, 2007; Van Overwalle, 2009; Van Overwalle and Baetens, 2009; Ross and Olson, 2010).

## Methods

### Participants

Two-hundred and thirty right-handed undergraduate and graduate university students (111 females, mean age ± s.d. = 23.02 ±2.65) participated in the study. Data from two participants with GQ-6 or trait elevation score beyond three deviations from the mean, respectively, were excluded from analyses, and thus data from 228 participants (109 females, mean age ± s.d. = 22.90 ± 2.57) were included in analyses. All of them were recruited through online advertisement. All participants gave informed consent and the protocols were approved by the Institutional Review Board of School of Medicine of Tsinghua University.

### Measures

#### Trait gratitude

The trait gratitude was measured by the GQ-6 (McCullough *et al.*, 2002), which is the most widely used scale for trait gratitude. The scale consists of six items, such as `I have so much in life to be thankful for’. Participants rated the items using a 7-point Likert scale from 1 = strongly disagree to 7 = strongly agree. The scale was reliable (α = 0.80).

#### Trait elevation

The trait elevation scale was adapted from Schnall *et al.*(2010). The scale consists of six items: ‘I often feel moved by moral behaviors reported in the news or happening around me’, ‘I often feel uplifted by moral behaviors reported in the news or happening around me’, ‘Whenever I saw or read about moral behaviors, I held optimistic view about humanity’, ‘I often have warm feeling in chest because of moral behaviors reported in the news or happening around me’, ‘Whenever I saw or read about moral behaviors, I wanted to help others’ and ‘Whenever I saw or read about moral behaviors, I wanted to become a better person’. Participants rated the items using a 7-point Likert scale from 1 = strongly disagree to 7 = strongly agree. The scale was reliable (α = 0.93). The correlations between the total score of this scale and scores of other relevant scales/variables (including state elevation) can be found in Supplementary Table S1.

### Image acquisition

Structural MRI was performed on a 3.0 T Philips Achieva 3.0 T TX system with a SENSE 8-channel head coil at the Center of Biomedical Engineering, Tsinghua University. T1-weighted structural images were acquired with TR of 8.2 ms, TE of 3.8 ms and flip angle of 8°. The SENSE factor was 2/1.5 for AP/RL, and the acquisition matrix was 256 mm × 256 mm. One hundred and sixty contiguous sagittal slices were acquired with voxel size of 1 mm × 1 mm × 1 mm.

### Image pre-processing

Images were preprocessed following the procedures described in the VBM tutorial by Ashburner (2010). T1-weighted images were pre-processed using Statistical Parametric Mapping (SPM) software (SPM8; Wellcome Department of Cognitive Neurology, London, United Kingdom; www.fil.ion.ucl.ac.uk/spm). For a better registration, the orientation and origin point was manually adjusted to match the template for each participant. The adjusted images were segmented into gray matter, white matter and cerebrospinal fluid images using the ‘New Segmentation’ module in SPM8. For better inter-subject alignment, the Diffeomorphic Anatomical Registration Through Exponentiated Lie (DARTEL) algorithm (Ashburner, 2007) in SPM8 was employed to normalize the segmented gray matter images to Montreal Neurological Institute (MNI) space (including resampling to 1.5 mm cubic isotropic voxels). Individual segmented gray matter images were nonlinearly warped to match the space of the DARTEL template and were modulated to preserve gray matter volumes. A Gaussian kernel of full width at half maximum (FWHM) equal to 4 mm was applied to smooth the normalized images. Previous studies showed that a 4 mm smoothing kernel is sufficient to correct for misalignment because of the increased accuracy of the DARTEL registration algorithm (Henley *et al.*, 2010; Shen and Sterr, 2013).

### VBM analysis

Statistical analyses were performed on pre-processed gray matter images using SPM8.

#### Identification of regions supporting trait gratitude and elevation respectively

In order to identify whether certain region supports trait gratitude and trait elevation, respectively, two voxel-wise generalized linear models (GLMs) were performed across the whole brain, with the GQ-6 (Model 1) or trait elevation score (Model 2), age, gender and total incranial volume (TIV) as covariates. A gray matter mask was created by binarizing SPM’s prior probability gray matter map at the threshold of 0.2 and was applied to the group-level analyses. In order to identify whether certain region supports trait gratitude, we conducted two contrasts (one positive correlation, the other negative) with GQ-6 score as covariate of interest, whereas age, gender and TIV as confounding covariates. Likewise, we conducted two other contrasts with trait elevation as covariate of interest and the other variables as confounding factors. Statistical maps were thresholded at *P_ucorr_* < 0.001 and clusters were considered as significant if passing a cluster-level threshold of *P* < 0.05 after familywise error correction. The cluster-level *P*-value was calculated with non-stationary extent correction (Hayasaka *et al.*, 2004). We further extracted the adjusted GMV of the peak voxel for the main results of trait gratitude and trait elevation, and conducted Pearson correlation analyses between corresponding self-report measure scores and them.

#### Identification of regions supporting trait gratitude and elevation distinctively

In order to identify whether certain region supports trait gratitude or trait elevation distinctively, a voxel-wise GLM (Model 3) was performed across the whole brain, with the GQ-6, trait elevation, age, gender and TIV as covariates. A gray matter mask was created by binarizing SPM’s prior probability gray matter map at the threshold of 0.2 and was applied to the group-level analyses. In order to identify whether certain region supports trait gratitude distinctively, we conducted two contrasts (one positive correlation, the other negative) with GQ-6 score as covariate of interest, whereas trait elevation, age, gender and TIV as confounding covariates. Likewise, we conducted two other contrasts with trait elevation as covariate of interest whereas the other variables as confounding factors. Statistical maps were thresholded at *P_ucorr_* < 0.001 and clusters were considered as significant if passing a cluster-level threshold of *P* < 0.05 after familywise error correction. The cluster-level *P*-value was calculated with non-stationary extent correction (Hayasaka *et al.*, 2004). We further extracted the adjusted GMV of the peak voxel for the main results of trait gratitude and trait elevation, and conducted Pearson correlation analyses between corresponding self-report measurement scores and them.

**Table 1:** Descriptive statistics of the self-report measures

	*N*	Minimum	Maximum	Mean	s.d.
Trait gratitude	228	22	42	34.45	4.64
Trait elevation	228	11	42	30.18	6.62

#### Identification of regions supporting both trait gratitude and elevation

In order to identify whether certain region supports both trait gratitude and elevation, statistical maps from Model 1 and Model 2 were thresholded at *P_ucorr_* < 0.001 and clusters were considered as significant if passing a cluster-level threshold of *P* < 0.05 after familywise error correction. The cluster-level *P*-value was calculated with non-stationary extent correction (Hayasaka *et al.*, 2004). One conjunction analysis was performed by multiplying both thresholded statistical maps obtained from positive correlation contrasts of the models, while another one with those obtained from negative correlation contrasts.

**Table 2:** Regions with GMV significantly correlated with trait gratitude and trait elevation without or with each other controlled in a whole-brain analysis. (*P* < 0.001 uncorrected at voxel level, cluster-level *P_FWE_* < 0.05)

Region	Direction of correlation	Cluster size (K)	Hemisphere	MNI coordinates			*Z*
				x	y	z	
**Trait gratitude, without controlling elevation**							
Cerebellum extending to fusiform gyrus	Positive	684	Left	−35	−39	−28	4.79
MOG extending to pSTS/TPJ (MTG & angular gyrus), BA39/19	Positive	225	Right	48	−80	14	4.26
**Trait elevation, without controlling gratitude**							
DLPFC (IFG & MFG), BA46/10	Negative	347	Left	−45	36	15	4.44
**Trait gratitude, with elevation controlled**							
Cerebellum extending to fusiform gyrus	Positive	1038	Left	−35	−39	−28	5.19
**Trait elevation, with gratitude controlled**							
DLPFC (IFG and MFG), BA46/10	Negative	453	Left	−42	48	6	4.56

MOG, middle occipital gyrus; pSTS, posterior superior temporal sulcus; MTG, middle temporal gyrus; TPJ, temporoparietal junction; DLPFC, dorsolateral prefrontal cortex; IFG, inferior frontal gyrus; MFG, middle frontal gyrus; BA, Brodmann area.

## Results

### Self-report measures

Table 1 presents descriptive statistics of the self-report measures. Pearson correlation analysis between the scores of trait gratitude and trait elevation was conducted. We found that they were significant positively correlated with each other, *r* = 0.27, *P* < 0.001. The correlations between trait gratitude/elevation and other relevant variables can be found in Supplementary Table S1.

### VBM results

#### Identification of regions supporting trait gratitude and elevation respectively

**Fig. 1:**
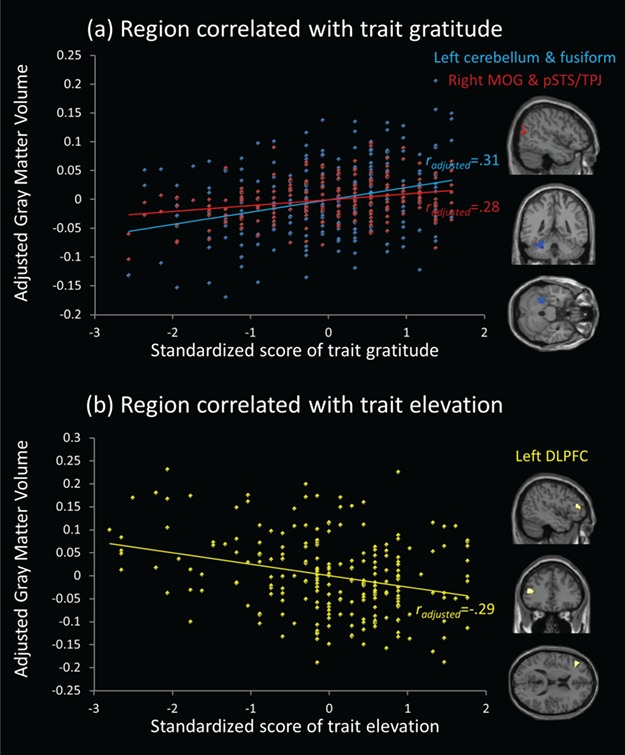
(**a**) A cluster in the left cerebellum and fusiform gyrus and a cluster in the right MOG and pSTS/TPJ were positively correlated with trait gratitude, with the adjusted correlation coefficient as 0.31 and 0.28, respectively. (**b**) A cluster in the left DLPFC was negatively correlated with trait elevation, with the adjusted correlation coefficient as −0.29.

Whole-brain VBM results of trait gratitude are presented in Table 2 and Figure 1A. The analysis demonstrated that trait gratitude was positively correlated with GMV in the left cerebellum extending to fusiform gyrus (peak MNI coordinate [−35–39 -28], Z = 4.79, *r_adjusted_* = 0.31, *P* < 0.001), and that in the right middle occipital gyrus (MOG) extending to pSTS/TPJ (peak MNI coordinate: [48–80 14], Z = 4.26, *r_adjusted_* = 0.28, *P* < 0.001). The results with positive affect controlled as covariate remained significant, which can be found in Supplementary Table S2.

Whole-brain VBM results of trait elevation are presented in Table 2 and Figure 1B. The analysis demonstrated that trait elevation was negatively correlated with GMV in the left DLPFC (peak MNI coordinate: [−45 36 15], Z = 4.44, *r_adjusted_* = −0.29, *P* < 0.001). The results with positive affect controlled as covariate remained significant, which can be found in Supplementary Table S2.

#### Identification of regions supporting trait gratitude and elevation distinctively

**Fig. 2:**
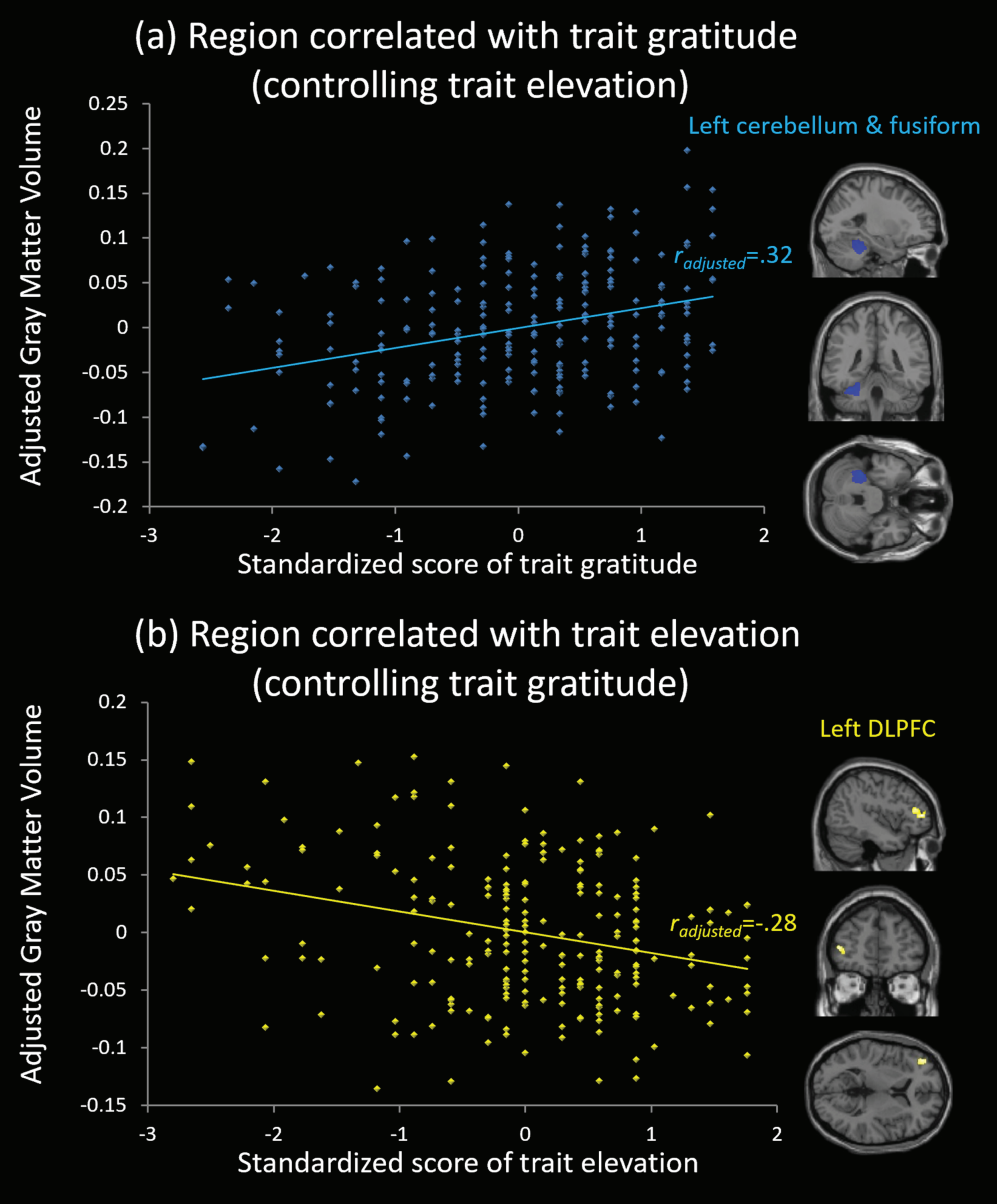
(**a**) A cluster in the left cerebellum and fusiform gyrus was positively correlated with trait gratitude when elevation was controlled, with the adjusted correlation coefficient as 0.32. (**b**) A cluster in the left DLPFC was negatively correlated with trait elevation when gratitude was controlled, with the adjusted correlation coefficient as −0.28.

Whole-brain VBM results of trait gratitude with elevation controlled are presented in Table 2 and Figure 2A. The analysis demonstrated that trait gratitude was positively correlated with GMV in the left cerebellum extending to fusiform gyrus (peak MNI coordinate [−35–39 -28], Z = 5.19, *r_adjusted_* = 0.32, *P* < 0.001). See above. The results with positive affect additionally controlled as covariate remained significant, which can be found in Supplementary Table S2.

Whole-brain VBM results of trait elevation with gratitude controlled are presented in Table 2 and Figure 2B. The analysis demonstrated that trait elevation was negatively correlated with GMV in the left DLPFC (peak MNI coordinate: [−42 48 6], Z = 4.56, *r_adjusted_* = −0.28, *P* < 0.001). The results with positive affect additionally controlled as covariate remained significant, which can be found in Supplementary Table S2.

#### Identification of regions supporting both trait gratitude and elevation

The conjunction analyses did not demonstrate any region supporting both trait gratitude and elevation.

## Discussion

In the present study, we found that trait gratitude was positively correlated with GMV in the left cerebellum extending to fusiform gyrus and the right MOG extending to pSTS/TPJ, while trait elevation was negatively correlated with that in the left DLPFC. While controlling each other, all the regions still held significant, except the right MOG and pSTS/TPJ. On the other hand, conjunction analyses did not show any region supporting both traits. These findings imply that trait gratitude and elevation have distinct neurostructural correlates, while the evidence is mixed that pSTS/TPJ may be the common correlates for them.

We found that trait gratitude was positively correlated with GMV in the left cerebellum extending to fusiform gyrus and the right MOG extending to pSTS/TPJ. These results were consistent with Zahn *et al*.’s (2013) findings that trait gratitude was correlated with posterior cortical volume, and also some fMRI studies showing that gratitude activated temporal cortex (Fox *et al*., 2015; Yu *et al*., 2018). According to the event–feature–emotion complexes (EFECs) model (Moll *et al*., 2005; Moll *et al*., 2008), moral cognitive phenomena emerge from the integration of content- and context-dependent representations in cortical–limbic networks, which rely on three main components: structured event knowledge, social perceptual and functional features, and central motive and emotional states. Among them, social perceptual and functional features are represented as context-independent knowledge in the anterior and posterior temporal cortex. As a moral emotion, gratitude relies much on visuo-spatial representations of morally salient scenes and a well-developed posterior cortical system may facilitate construction of detailed scenes, which could play an important role for experiencing gratitude (Zahn *et al*., 2013). It’s been speculated that posterior cortical networks are involved in visual imagery, although not necessary for moral emotions, may play a supportive role in allowing for more lively and detailed scenic representations of social behavior and hence more intense emotional experience when visuo-spatial memories or mental models are required (Moll *et al*., 2008; Zahn *et al*., 2013). A previous study has shown a high correlation between visual imagery, autobiographical episodic retrieval and emotional intensity (Zahn *et al*., 2008).

More importantly, the relationship between trait gratitude and GMV in the left cerebellum and fusiform gyrus remained significant after elevation was controlled, which indicates that this region distinctively supports gratitude rather than common to all other-praising emotions. Interestingly, Zahn *et al*. (2013) found that proneness to gratitude was positively correlated with GMV in the inferior temporal gyrus, disruption of the integrity of which is associated with both acquired (Barton, 2008) and developmental prosopagnosia (Dinkelacker *et al*., 2011), while we found trait gratitude was positively correlated with the fusiform gyrus, a region widely reported to be involved in face perception (Kanwisher *et al*., 1997; McCarthy *et al*., 1997; Rossion *et al*., 2003; Kanwisher and Yovel, 2006). Results from both studies suggest that facial imagery is important for experiencing gratitude. Stronger ability in face perception may enable individuals to remember more faces, including those of benefactors, and thus remember more people to be grateful for. This is consistent with the evolutionary theory of gratitude as an adaptation for reciprocal altruism (McCullough et al., 2008). From the perspective of evolutionary biology, the ability to recognize many different previous interactants is invaluable for an organism to extend the range of stable cooperation (Axelrod and Hamilton, 1981). On the other hand, the fusiform gyrus is also associated with other social cognitions, such as processing emotional pictures of social complexity (Geday *et al*., 2003), decoding communicative intentions (Wang *et al*., 2006) and storing general information about people (Schultz *et al*., 2003). These results are consistent with previous argument that benevolent intention is critical in inducing gratitude (McConnell, 1993; Yu *et al*., 2017), the notion of gratitude as other-praising emotion and also previous findings that the motivation of gratitude is more relationship-based compared to elevation and other positive emotions (Algoe and Haidt, 2009; Algoe, 2012). Previous research has consistently found that the level of benevolent intention of the benefactor predicted the intensity of gratitude (Tesser *et al*., 1968; Wood *et al*., 2008; Yu *et al*., 2017). In addition to the fusiform gyrus, we also found GMV in the left cerebellum showed a positive correlation with trait gratitude distinctively. A meta-analysis (Van Overwalle *et al*., 2014) explored the role of the cerebellum in social cognition, and found that abstraction in mentalizing (e.g. projecting oneself into the future and recalling the autobiographical past, thinking about traits and stereotypes) strongly involved the cerebellum, which indicates that increased difficulty or cognitive load involved in abstraction may cause increased cerebellar activity. Combining the findings on the relationship between trait gratitude and the fusiform gyrus, bigger cerebellum may enable stronger ability in recalling autobiographical past, remembering more grateful experiences and thus experiencing gratitude more frequently.

We also found that trait elevation was negatively correlated with GMV in the left DLPFC. More importantly, these results remained significant even after trait gratitude was controlled, which indicates that left DLPFC distinctively supports elevation. According to Greene’s dual-process theory of moral judgement (Greene, 2007, 2009), deontological moral reasoning is subserved by VMPFC, while utilitarian moral reasoning is subserved by DLPFC, which is based on the view that DLPFC is involved in cognitive control (Miller and Cohen, 2001). According to the theory, people with smaller DLPFC should show weaker ability in utilitarian moral reasoning. Previous research did show that elevation decreased permissiveness for deontological violations in moral judgment (Strohminger *et al*., 2011). According to this view, individuals with smaller DLPFC may be weaker in utilitarian moral reasoning, more focused on admiration of the praiseworthy behavior itself rather than thinking about its outcome, and are more prone to feel elevation. An alternative interpretation also based on the cognitive control view of DLPFC is related to the intuitive view of prosociality. According to this view, prosociality is intuitive while selfish behavior and self-interest maximization are out of the regulation of cognitive control (Rand *et al*., 2012; Zaki and Mitchell, 2013; Yamagishi *et al*., 2016). Some previous neuroimaging studies supported this view, showing that the cortical thickness in the DLPFC was negatively correlated with giving in dictator game and strategic reasoning behavior (Yamagishi *et al*., 2016). Considering previous findings that the motivation of elevation is more morality−/prosociality-based compared to gratitude, smaller left DLPFC may contribute to trait elevation through weaker ability in self-interest maximization and suppressing intuitive drive for prosociality. There is an alternative model to the function of DLPFC. According to Grafman’s (1995) structured event complex model and Moll *et al*.’s (2005) EFECs model, prefrontal cortex serves to store long-term goals and multi-state event complexes and is implicated in integrating separate cognitive operations to achieve a superordinate behavioral goal. According to the models, less predictable event sequences/action tendencies are represented in the DLPFC. Therefore, having less well-developed representations of sequential action/event contexts may make people more likely to generalize experiences of elevation to more different types of action contexts (Zahn *et al*., 2013).

It is noteworthy that the correlation between trait gratitude and GMV in the MOG extending to pSTS/TPJ did not hold significant after elevation was controlled, indicating that the cluster did not distinctively support trait gratitude, and may be the common neural correlates to both gratitude and elevation. Previous fMRI studies on gratitude (Fox *et al*., 2015; Yu *et al*., 2018) also found that pSTS/TPJ was activated, and it was thought to be associated with intentionality processing. An abundance of research has shown that pSTS/TPJ is associated with theory of mind and intention inference (Frith and Frith, 2003; Gallagher and Frith, 2003; Singer, 2006; Carrington and Bailey, 2009; Van Overwalle, 2009; Van Overwalle and Baetens, 2009). It is thought that complex social emotions, such as jealousy, pride, embarrassment and guilt, often imply awareness of another person’s attitude to oneself and that of the self in relation to other people, and thus are likely to involve the mentalizing system (Blakemore *et al*., 2004). Empirical neuroimaging studies on gratitude (Fox *et al*., 2015; Yu *et al*., 2018), embarrassment (Berthoz *et al*., 2002) and forgiveness (Farrow *et al*., 2001) supported this view. Because both gratitude and elevation are other-praising emotions involved in processing social relationship between self and others, bigger pSTS/TPJ may support both gratitude and elevation. On the other hand, pSTS is a key region for sensory-driven social attention to salient stimuli (Sui *et al*., 2013) and storing social perceptual and functional features (Allison *et al*., 2000; Frith, 2001) extracted from facial expression, gaze, prosody, body posture and gestures (Moll *et al*., 2005). As speculated by Zahn *et al*. (2013), a well-developed posterior cortical system may facilitate construction of detailed scenes with more elaborate sensory imagery, which is important for experiencing gratitude. Bigger pSTS/TPJ may contribute to both gratitude and elevation by facilitating construction of detailed scenes. However, because the conjunction analyses did not show any significant region correlating with both traits, the conclusion that pSTS/TPJ supports both trait gratitude and elevation is preliminary and needs further reproduction.

Intriguingly, our study did not show a significant association between trait gratitude/elevation and regions associated with reward processing. It seems counterintuitive, because previous research has shown that intensity of positive emotions are modulated or predicted by activity in those regions, such as MPFC and OFC (Fox *et al*., 2015; Ashar *et al*., 2017). In addition, MPFC has also been found activated when experiencing gratitude in most fMRI studies (Fox *et al*., 2015; Kini *et al.*, 2016; Karns *et al*., 2017; Wang *et al*., 2017; Yu *et al*., 2017; Yu *et al*., 2018). Considering affect on the trait level reflects the frequency rather than the intensity of emotions, however, it may be possible that the frequency of positive affective experience is less associated with momentary reward processing. On the other hand, VBM is not sensitive at detecting some regions associated with reward processing, such as hypothalamus, which was found activated in experiencing gratitude (Zahn *et al*., 2008).

In summary, the current study provides the first piece of evidence that the two important other-praising affects (i.e. trait gratitude and trait elevation) have distinct neurostructural correlates and may be supported commonly by pSTS/TPJ. The results are consistent with previous literature showing that gratitude and elevation are distinct from each other and other positive emotions (Algoe and Haidt, 2009; Siegel *et al*., 2014; Stellar *et al*., 2017). One limitation of the present study is that the sample was mainly recruited from undergraduate and graduate students, which may limit the generalizability of our results. In addition, the present study are largely correlational, the causal relationship between affective traits and certain neural regions remained unclear. Future study should apply longitudinal design in a more age-ranged sample to address the issue. Future research should also include measures of potential intermediating variables, such as face processing, intentionality inference and cognitive control, to examine the exact relationship between these affective traits and corresponding neural regions.

## 

*Conflict of interest*. None declared.

## Funding

This work was supported by Economic and Social Research Council (ES/K013424/1); National Nature Science Foundations of China (No. 31170973 and No. 31471001); Tsinghua University Initiative Scientific Research Program (to J.S.); Positive Psychology Research Foundation of China (0020344, 2015-01-29).

## Supplementary Material

Supplementary Data
