# Supplementary material for: Praising others differently: neuroanatomical correlates to individual differences in trait gratitude and elevation
Source: Soc Cogn Affect Neurosci. 2018 Oct 23;13(12):1225–34. doi: 10.1093/scan/nsy093 (PMC6277740; doi:10.1093/scan/nsy093)
Supplement: Supplementary Data [file nsy093_supp.docx]

**Supplementary Materials**

**Praising others differently: Neuroanatomical correlates to individual differences in trait gratitude and elevation**

Guanmin Liu^a,*^, Guang Zeng^a,*^, Fei Wang^a^, Pia Rotshtein^b^, Kaiping Peng^a^, Jie Sui^c^

Author Note

^a^Department of Psychology, Tsinghua University, Beijing, 100084, China

^b^School of Psychology, University of Birmingham, Birmingham, B15 2TT, UK

^c^Department of Psychology, University of Bath, Bath, BA2 7AY, UK

Correspondence should be addressed to Kaiping Peng, Department of Psychology, Tsinghua University, Beijing 100084, China. Email: [pengkp@mail.tsinghua.edu.cn](mailto:pengkp@mail.tsinghua.edu.cn); or Jie Sui, Department of Psychology, University of Bath, Bath, BA2 7AY, UK. Email: [j.sui@bath.ac.uk](mailto:j.sui@bath.ac.uk)

Present address: Guanmin Liu, Center for Healthy Minds, University of Wisconsin-Madison, Madison, WI 53703, USA

^*^ Guanmin Liu and Guang Zeng contributed equally to the work.

**Supplementary Methods**

**Supplementary Measures**

*State elevation* The state elevation scale developed by Vianello et al. (2010; study 1) was applied to assess participants’ state elevation level after reading a piece of news where the protagonist risked her life saving others. The scale consists of 8 items. Participants rated how strong they felt on each item using a 7-point Likert scale from 1= *not at all* to 7=*extremely strongly*.

*Positive affect and negative affect* The Positive and Negative Affect Schedule (PANAS; Watson et al., 1988) was applied to assess participants’ general tendency to experience positive affect and negative affect. The scale consists of 10 positive affective adjectives and 10 negative affective adjectives. Participants rated how frequently they have felt each emotion recently using a 5-point Likert scale from 1= *very randomly or not at all* to 5=*extremely frequently*. Note that two out of the 228 participants did not finish the scale.

*Reward responsiveness* The Reward Responsiveness Scale (Van den Berg et al., 2010) was applied to assess participants’ tendency to reward responsiveness. The scale consists of 8 items. Participants rated the extent to which they agreed with each item using a 4-point Likert scale from 1= *strongly disagree* to 4=*strongly agree*.

*Self-esteem* The Rosenberg Self-esteem Scale (Rosenberg, 1965) was applied to assess participants’ self-esteem. The scale consists of 10 items. Participants rated the extent to which they agreed with each item using a 4-point Likert scale from 1= *strongly disagree* to 4=*strongly agree*.

*Big Five personality* The Big Five Inventory (BFI) (John et al., 1991) was applied to assess participants’ big five personality. The scale consists of 44 items, measuring the five dimensions of Big Five personality: openness, conscientiousness, extraversion, agreeableness and neuroticism. Participants rated the extent to which they agreed with each item using a 5-point Likert scale from 1= *strongly disagree* to 5=*strongly agree*. Note that two out of the 228 participants did not finish these scales.

**Supplementary VBM Analysis**

Statistical analyses were performed on pre-processed gray matter images using SPM8 with the same methods described in the main text. All the supplementary models performed here are the same as Model 1, 2 and 3 described in the main text, the only difference being that positive affect was added as covariant in all the models. Note that the missing two participants’ positive affect scores were substituted by group’s mean value.

**Supplementary Results**

**Correlation between trait gratitude/elevation and relevant variables**

**Supplementary Table S1.** Correlations between trait gratitude/elevation and relevant variables.

|  | 1 | 2 | 3 | 4 | 5 | 6 | 7 | 8 | 9 | 10 | 11 | 12 |
| --- | --- | --- | --- | --- | --- | --- | --- | --- | --- | --- | --- | --- |
| 1 Trait Gratitude | - |  |  |  |  |  |  |  |  |  |  |  |
| 2 Trait Elevation | **.27^**^** | - |  |  |  |  |  |  |  |  |  |  |
| 3 State Elevation | **.17^*^** | **.58^**^** | - |  |  |  |  |  |  |  |  |  |
| 4 Positive Affect | **.29^**^** | **.31^**^** | **.37^**^** | - |  |  |  |  |  |  |  |  |
| 5 Negative Affect | **-.18^**^** | -.11 | **-.07** | **-.14^*^** | - |  |  |  |  |  |  |  |
| 6 Reward Responsiveness | **.38^**^** | .08 | 0.12 | **.36^**^** | **-.17^*^** | - |  |  |  |  |  |  |
| 7 Self-Esteem | **.50^**^** | **.31^**^** | **.22^**^** | **.48^**^** | **-.41^**^** | **.39^**^** | - |  |  |  |  |  |
| 8 BFI-Openness | **.28^**^** | **.14^*^** | .07 | **.32^**^** | **-.14^*^** | **.27^**^** | **.40^**^** | - |  |  |  |  |
| 9 BFI-Conscientiousness | **.17^*^** | .11 | **.15^*^** | **.43^**^** | **-.50^**^** | **.33^**^** | **.43^**^** | .12 | - |  |  |  |
| 10 BFI-Extraversion | **.32^**^** | **.23^**^** | **.21^**^** | **.53^**^** | **-.37^**^** | **.36^**^** | **.52^**^** | **.48^**^** | **.34^**^** | - |  |  |
| 11 BFI-Agreeableness | **.30^**^** | **.30^**^** | **.28^**^** | **.26^**^** | **-.33^**^** | **.17^*^** | **.27^**^** | **.20^**^** | **.34^**^** | **.27^**^** | - |  |
| 12 BFI-Neuroticism | **-.20^**^** | **-.15^**^** | **-.20^**^** | **-.34^**^** | **.58^**^** | -.09 | **-.44^**^** | **-.23^**^** | **-.55^**^** | **-.46^**^** | **-.45^**^** | - |

^**^*p* < .01; ^*^*p* < .05.

**Neuroimaging results with positive affect controlled**

**Supplementary Table S2.** Regions with gray matter volume (GMV) significantly correlated with trait gratitude and trait elevation, controlling positive affect (PA), without or with each other controlled in a whole-brain analysis. (*p*<.001 uncorrected at voxel level, cluster-level *p_FWE_*<.05).

| Region | Direction of Correlation | Cluster Size (K) | Hemisphere | MNI coordinates | | | *Z* |
| --- | --- | --- | --- | --- | --- | --- | --- |
|  |  |  |  | x | y | z |  |
| **Trait gratitude, controlling PA but not elevation** |  |  |  |  |  |  |  |
| Cerebellum extending to fusiform gyrus | Positive | 643 | Left | -35 | -39 | -28 | 4.81 |
| MOG extending to pSTS/TPJ (MTG & angular gyrus), BA39/19 | Positive | 263 | Right | 35 | -83 | 30 | 4.02 |
| **Trait elevation, controlling PA but not gratitude** |  |  |  |  |  |  |  |
| DLPFC (IFG & MFG), BA46/10 | Negative | 312 | Left | -47 | 36 | 15 | 4.46 |
| MPFC extending to ACC, BA10^*^ | Negative | 116 | Left/Right | 0 | 52 | 6 | 4.96 |
| **Trait gratitude, with elevation and PA controlled** |  |  |  |  |  |  |  |
| Cerebellum extending to fusiform gyrus | Positive | 905 | Left | -35 | -39 | -28 | 5.11 |
| **Trait elevation, with gratitude and PA controlled** |  |  |  |  |  |  |  |
| DLPFC (IFG & MFG), BA46/10 | Negative | 375 | Left | -47 | 36 | 15 | 4.43 |

^*^ We did not discuss this cluster in the main text, because it was not significant when positive affect was not controlled as in the main analyses.

MOG, middle occipital gyrus; pSTS, posterior superior temporal sulcus; MTG, middle temporal gyrus; TPJ, temporoparietal junction; DLPFC, dorsolateral prefrontal cortex; IFG, inferior frontal gyrus; MFG, middle frontal gyrus; MPFC, medial prefrontal cortex; ACC, anterior cingulate cortex; BA, Brodmann area.

**Supplementary References**

John, O.P., Donahue, E.M., Kentle, R.L. (1991). *The big five inventory—versions 4a and 54*. Berkeley, CA: University of California, Berkeley, Institute of Personality and Social Research.

Rosenberg, M. (1965). *Society and the adolescent self-image*. Princeton, NJ: Princeton university press.

Van den Berg, I., Franken, I.H., Muris, P. (2010). A new scale for measuring reward responsiveness. *Frontiers in psychology*, **1**, 239.

Vianello, M., Galliani, E.M., Haidt, J. (2010). Elevation at work: The effects of leaders’ moral excellence. *The Journal of Positive Psychology*, **5**(5), 390-411.

Watson, D., Clark, L.A., Tellegen, A. (1988). Development and validation of brief measures of positive and negative affect: the PANAS scales. *Journal of personality and social psychology*, **54**(6), 1063-70.
